# 

**Published:** 1858-07

**Authors:** 


### Books Received.

"Institutes of Medicine, by Martyn Paine, A. M., M. D., L. L. D.," &c.

"Medical & Physiological Commentaries," by the same author, from whom, they have been received.

We have also received "Nature and Art in the cure of Disease," and, "Mind and Matter or Physiological Enquiries," from Messrs. S. S. & W. Wood, New York.

These books will be reviewed for the next number of the Journal.

